# Xerostomia: From Pharmacological Treatments to Traditional Medicine—An Overview on the Possible Clinical Management and Prevention Using Systemic Approaches

**DOI:** 10.3390/curroncol30050336

**Published:** 2023-04-24

**Authors:** Luigi Sardellitti, Antonella Bortone, Enrica Filigheddu, Francesca Serralutzu, Egle Patrizia Milia

**Affiliations:** 1 Department of Medicine, Surgery and Pharmacy, University of Sassari, 07100 Sassari, Italy; l.sardellitti@studenti.uniss.it; 2 Dental Unit, Head and Neck Department, Azienda Ospedaliero Universitaria, 07100 Sassari, Italy; antonella.bortone@aouss.it (A.B.); enrica.filigheddu@aouss.it (E.F.); 3 Institute for Animal Production Systems in the Mediterranean Environment (ISPAAM)-Section of Sassari, 07100 Sassari, Italy; francesca.serralutzu@cnr.it

**Keywords:** dry mouth, head and neck cancer, radiotherapy, oral mucositis, oral diseases, medicinal herbs

## Abstract

Despite high incidence rates and severe complications, the management of xerostomia lacks clinical guidelines. The aim of this overview was to summarize the clinical experience derived from the last 10 years of treatments and prevention using systemic compounds. Results showed that the cytoprotective drug amifostine, and its antioxidant agents, are the most discussed as preventive agents of xerostomia in head and neck cancer (HNC) patients. In the presence of the disease, the pharmacological treatments have been mainly directed to stimulate secretion of the damaged salivary glands, or to counteract a decreased capacity of the antioxidant system, in view of an increasing of reactive oxygen species (ROS). However, the data demonstrated low ability of the drugs, together with a great number of side effects, which strongly limit their use. Concerning traditional medicine (TM), valid clinical trials are so limited that neither the efficacy nor the absence of interferences to concomitant chemical therapies can be validated. Consequently, the management of xerostomia and its devastating complications remain a very significant void in daily clinical practice.

## 1. Introduction

Saliva is an important defense in maintaining well-being and oral health.

Saliva is an exocrine solution, consisting of 99% water. The remaining 1% consists of a variety of electrolytes and proteins, which are responsible for the various functions including speech, swallowing, and tasting [1]. Its enzymes start the digestion of starches and fats in the mouth, and other salivary components, such as the epidermal growth factors, promote tissue growth, cell differentiation, and allow wound-healing [2]. The antibacterial, antifungal, and antiviral agents in salivary fluid balance the oral biofilm, preventing dysbiosis, while the mineral components maintain the integrity of teeth, antagonizing demineralization processes [3]. Additionally, the salivary glycoproteins, mainly represented by mucins, together with the electrolytes, protect the mucosal structures from mechanical, microbial, and chemical injury. However, to fulfil all these roles, a continuous flow and a balanced composition of saliva is necessary in the oral cavity.

About 90% of the salivary secretion derives from the submandibular, the parotid, and the sublingual glands, which are under the control of the autonomic nervous system. Minor salivary glands, located at the labial, lingual, buccal, and palatal surfaces of the mouth, allow the production of the remaining 10% of the fluid. In healthy individuals, saliva production ranges from 0.5 to 1.5 L per day [4,5]. Normal salivary output at rest, without exogenous or pharmacological stimulation, is a small, continuous flow, in the form of a film that covers, moisturizes, and lubricates the oral tissues, maintaining oral health, while reflex saliva is produced in reply to sensory stimulation related to smelling, tasting, and chewing. All these stimulate the medulla salivary center in the central nervous system (CNS), which, in turn, stimulates the salivary gland nerves in saliva production [6].

Over the course of life, the function of salivary glands can be frequently impaired. Xerostomia is the term used to describe the subjective symptoms of dry mouth, deriving from a lack of saliva. It is generally manifested when the fluid is reduced to 40–50%, in comparison to the normal production, or if there are changes in its composition [7]. Xerostomia occurs when resting saliva is lowered to ≤0.1 mL/min, and stimulated saliva is ≤0.5 mL/min [4]. The incidence of xerostomia occurs in about 30% of the population aged 65+ years [8], with a prevalence in the female population. Furthermore, the incidence increases to 61% in the aging population, in view of comorbidity and poly-pharmacological treatments [9].

Autoimmune conditions, such as Sjögren’s Syndrome, and rheumatic and dysmetabolic diseases, such as diabetes and the hepatitis C virus, in addition to chronic use of more than 400 medications, are among the more recurrent causes of saliva alteration [10]. Drugs include antidepressants, antihypertensives, anticancer agents, opiates, bronchodilators, proton pump inhibitors, antipsychotics, antihistamines, diuretics, and others [5]. Further, radiation therapy for HNC is the most prevalent cause of salivary dysfunctions [5,11,12]. Several studies demonstrated that HNC radiation affects the major and minor salivary glands, contributing to a temporary or permanent impairment of the secretion for up to 40% of the patients [13]. Additionally, very recently, the novel SARS-CoV-2 infection was reported to have caused the development of xerostomia in 45.9% of the infected subjects [14].

Independent of causes, the most common symptom deriving from saliva alterations is dry mouth [15]. The feeling is related to a poor protection of the oral tissues offered by the altered saliva. This situation leads the mouth to undergo ulcerations, aphthosis, mucositis, and infections, with a general sensation of pain and/or a widespread burning sensation (burning mouth). Burning is mainly localized at the tongue, together with the hard palate, due to a greater evaporation of the saliva in comparison to the other regions of the mouth. Inadequate lubrication and protection also involve the pharynx mucosa, sometimes impairing speech and inducing cough. Sleep disturbances are also recurrent in xerostomia, due to the need to humidify the mucosa during the night; at which time, the saliva secretion is further reduced because of the circadian rhythm [2,16]. Furthermore, chewing and swallowing are negatively affected by the lower saliva, forcing patients to take continuous sips of water to facilitate food transit. Taste disfunction also affects the production of saliva [6]. It mostly impairs neoplastic patients suffering from xerostomia, negatively impacting their survival by causing psychological anxiety and malnutrition. The latter is one of the major factors underlying the high morbidity experienced by these patients [17].

Additionally, the lowered pH of the viscous saliva leads to a bacterial shift that can occur in the oral biofilm, leading to the development of dysbiosis. This represents further opportunities to develop gingivitis, caries, and mucositis, with oral candidiasis in immunocompromised subjects being frequently represented [3]. Thus, xerostomia not only diminishes the quality of life (QoL) in cancer patients, but also poses a major new health problem for them.

The management of xerostomia remains a significant clinical challenge. Treatments have been mainly directed to increase the saliva flow using pharmacological treatments, while local salivary substitutes have been used to relieve the sensation of dryness and the compromised oral functions. Furthermore, TM and complementary medicine (CM) have been evaluated, based on the increasing demand of their services, sustained by the WHO (2003-05 and 2011-23). However, in any case, the clinical conclusions and benefits of such therapeutic proposals appear mostly ambiguous [10,15]. Additionally, the compromised patients’ health and medication intake make the gamut of choices very narrow [18]. Therefore, an update on this topic is necessary to assess the current efficacy of the treatments and provide therapeutic recommendations based on the evidence.

Giving the above considerations and the need to have clinical indications in the management of xerostomia, the present review examines the existing data of the last 10 years to provide an update in the prevention and therapy of the disease.

The overview aims to focus on the following research questions:

(1) What approaches are there to prevent xerostomia in predisposed patients?
(2) Which systemic drugs are effective in antagonizing the disease?
(3) Is TM a valid method and an alternative to chemicals in the treatment of the disease?

With this purpose, a search for the existing literature published between 1 January 2012 and 30 January 2021 was performed in the PubMed, EMBASE, Web of Science, ScienceDirect, and Google Scholar databases, using specific keyword search terms.

## 2. Results

### 2.1. Systemic Chemical Treatments

The literature discusses a wide range of pharmacological interventions for salivary disfunction, particularly regarding patients receiving radiotherapy for HNC, and patients suffering from Sjögren’s Syndrome (Table 1).

### 2.2. Para-Sympathomimetic Drugs

Para-sympathomimetic drugs stimulate salivary secretion by means of the parasympathetic nervous system [15,26]. Activation of the parasympathetic fibers leads to saliva flow mediated by acetylcholine-induced activation of acinar cell muscarinic acetylcholine receptors (mAChRs). This, in turn, activates the release of G proteins from the Gq family [27], which mediate an increase in intracellular calcium levels and the opening of calcium-activated Cl^-^ and K^+^ channels [28]. Five molecularly distinct members (M1-M5) compose the mAChR family, based on their G protein-coupling profiles. Although the M3 receptor subtype plays a key role, pharmacological and biochemical studies suggest that M1 and M5 mAChRs may also have an influence on salivary output [29,30]. Identification of specific mAChR subtypes mediating salivary secretion is hopeful in the treatment of xerostomia. Conversely, little evidence can support the use of non-selective para-sympathomimetic drugs to resolve the lack of saliva in cases of dry mouth.

Pilocarpine hydrochloride (a choline ester) has been the most common para-sympathomimetic used in cases of dry mouth. Mostly, pilocarpine has been tested as an agent in the prevention and cure of xerostomia after radiotherapy for HNC, and in Sjogren’s patients [31]. In the Cochrane database, Davis and colleagues [19,20,26] reviewed the clinical effectiveness and toxicity of pilocarpine. The drug was administered in different forms (i.e., tablets, mouthwash) and doses (2.5 to 10 mg up to three times a day), concluding that pilocarpine can be effective, even if statistically significant differences comparing the treatment to placebo groups remain unclear [32]. Although the response likely depends on several factors (i.e., degree of salivary gland damage, medical problems, concomitant treatments), little evidence supports the use of pilocarpine in radiation-induced xerostomia. The most common side effects were headache (15%), urinary frequency (14%), vasodilatation (12%), dizziness (10%), dyspepsia (10%), nausea (8%), asthenia (8%), and diarrhea (5%), and their frequency was also the predominant reason for withdrawing patients from the studies.

Two randomized control trials evaluated cevimeline hydrochloride as a para-sympathomimetic drug in xerostomia [21,33]. Results suggested it was quite effective [33].

Bethanechol, a choline ester with muscarinic properties, was also studied in HNC cancer patients after irradiation [22]. Despite the great number of side effects (i.e., hyperthyroidism, peptic ulcer, asthma, bradycardia, hypotension, coronary diseases, epilepsy, Parkinsonism), bethanechol seemed to increase the basal salivary flow rate, in comparison to artificial saliva [15]. However, the side effects really limit its use [18].

### 2.3. Para-Sympatholytic Drugs

Para-sympatholytic drugs work in opposition to para-sympathomimetics inhibiting the secretion of saliva fluid. During radiotherapy, the inhibition of salivary secretion in animal testing evidenced it might protect the salivary glands from later damage [26].

### 2.4. Cytoprotective Agents

Cytoprotective agents can be administered before or after cancer therapy, with the intent to prevent or reduce damage or toxicity to the normal tissues, without compromising therapeutic efficacy. Among them, amifostine is an organic thiophosphate that is indicated against the harmful effects of radiation or chemotherapy, including acute or late xerostomia. The cytoprotective mechanism involves free radical scavenging, DNA protection and repair acceleration, and induction of cellular hypoxia. Amifostine represents the inactive prodrug that becomes active after being dephosphorylated in cell plasma membranes. The active metabolite, WR-1065, scavenges free radicals by accumulating in the normal tissues, in which the drug protects cellular membranes and DNA from dysfunctions [34,35].

The capacity of amifostine was evaluated in several studies. Some of them reported a reduction of xerostomia by radiation therapy [34,36]. Nevertheless, no clear evidence showed its beneficial effect when it was compared to normal saline IV as placebo. Conversely, after radiotherapy, differences in the worst grade of skin and mucosal toxicity between the arms were demonstrated in HNC patients, together with an increased risk of vomiting [34,37], hypotension [38], nausea [39], and allergic responses [34,37,39,40]. Finally, controversy exists regarding whether amifostine might reduce the efficacy of cancer treatment, influencing the overall survival, progression-free survival, disease-free survival, and, if it is useful, QoL.

### 2.5. Antioxidant Agents

The use of antioxidant agents in xerostomia is related to the fact that oxidative stress is involved in the issue [41]. Oxidation is implicated in the development of xerostomia in cases of Sjögren’s Syndrome, radiotherapy patients, and systemic sclerosis. CoQ10 is one of the most studied agents, as it is required in the direct and indirect protection of the cell membranes, ATP production, and secretory function [42,43,44]. In Sjögren’s Syndrome, several studies proved the efficiency of CoQ10 precursors by administering capsules of ubiquinone/ubiquinol in comparison to placebo [23,24]. A general increase in salivary production was observed in the patients, even if ubiquinol showed higher efficacy in comparison to ubiquinone [23].

In radiotherapy patients, the well-known free radical scavenger activity of α-tocopherol (Vitamin E) was assayed. Together with the recovery of important salivary parameters (i.e., pH, potassium levels, and amylase activity), a significant improvement in the flow rate was found [45]. Further, the administration of 100 IU of Vitamin E + 500 mg of Vitamin C during the radiotherapy cycles resulted in greater improvement in VAS scores in comparison to the control group [25].

Additionally, natural carotenoids have been assessed in xerostomia. Among them, lycopene was intensively studied, due to its proven activity in decreasing serum lipid peroxidation and low-density lipoprotein oxidation [44]. The studies evidenced that lycopene-enriched virgin olive oil has higher efficacy in the relief of dry mouth in comparison to the isolated compound [46,47]. Furthermore, the correlation between lycopene, DNA methylation, and inflammation warrants further investigation, because it may impact health outcomes in HNC survivors [48].

### 2.6. Biological Agents

Biological agents have been proposed in Sjogren’s Syndrome patients, with the intent to antagonize the immunological disfunction evidenced by the infiltration of lymphocytic in exocrine and non-exocrine epithelia [49]. Among the biological compounds, rituximab was the first agent assessed in the field. It is a chimeric mouse/human monoclonal anti-CD20 antibody binding to a CD20 B cell surface antigen. Rituximab induces a depletion of the B cell. A double-blind, randomized, placebo-controlled study [50] demonstrated an increase in stimulated and unstimulated whole saliva flow lacrimal gland functions, and a decrease in extra-glandular manifestations after rituximab treatment. However, at the 48-week point of the study, the beneficial effects were lost and no significant differences in saliva was observed between the study groups. Regarding the VAS scores, only ocular dryness showed a significant improvement at the 48-week control point, while patients experienced common side effects, such as a serum sickness-like disease and several infections. Other biological agents, such as epratuzumab, belimumab, ianalumab, and baminercept, have been evaluated in reducing dry mouth in Sjögren’s Syndrome. Epratuzumab, a humanized anti-CD22 monoclonal antibody, has been associated with an improvement in lacrimal gland function and unstimulated whole saliva flow rate. However, important side effects were found, among which were acute infusion reactions and infections [51,52]. Belimumab is a human IgG1 λ mAb-targeting B lymphocyte stimulator, with the effect to lead an inhibition of the B cells. The administration of 10 mg/kg monthly dose did not show any changes in unstimulated saliva flow, while it caused the development of infections, among which, pneumococcal meningitis was the most serious [53]. Further, ianalumab, a monoclonal antibody (VAY736), and baminercept, a lymphotoxin-β receptor fusion protein, have been studied. However, the results demonstrated low efficacy associated to toxicity [54,55].

T-cell-targeting drugs have also been hypothesized in the relief of Sjögren’s Syndrome. Among them, abatacept is a human fusion molecule that blocks the interaction between CD80/86 (in APC) and CD28 ligand on T-cell surfaces, inactivating the cells’ proliferation and the production of cytokines. However, the results of the advantage of abatacept are controversial in the trials, as some studies showed a reduction of inflammatory markers in the salivary glands, while others described no changes in salivary and lacrimal gland functions [56,57] Furthermore, lymphocytic foci and lupus-like cutaneous lesions were reported as side effects of abatacept, obviously limiting its evaluation [55].

Regarding the humanized monoclonal antibody efalizumab, no increase in salivation was found by its use, while the risk of multifocal leukoencephalopathies imposed the withdrawal of the agent from the market [58]. Drugs such as infliximab (TNF family), etanercept (TNF family), tocilizumab (IL-6), and anakinra (IL-1), have also been evaluated as cytokine-targeted therapies in Sjögren’s Syndrome. Results showed no significant changes in salivary or lacrimal gland production between the administered groups and the placebo groups [54].

Further, interferon alpha has been studied in Sjögren’s Syndrome. Interferons are cytokines, and antiviral and regulatory proteins in inflammation and immune response. It is thought that interferon alpha may upregulate aquaporins 5, which is abnormally distributed in salivary and lacrimal glands in Sjögren’s Syndrome patients [59]. A double-blind placebo-controlled study [60], and other clinical trials [59], showed an improvement in oral and ocular dryness. Other studies found an increase in unstimulated salivary flow compared to placebo [18]. However, a higher percentage of gastrointestinal adverse effects render its use very questionable [55].

### 2.7. Traditional Medicine

WHO defines TM as a “medicine of long history that is the sum of the knowledge, skill, and practices based on the theories, beliefs, and experiences of different cultures, whether explicable or not, used in the maintenance of health as well as in the prevention, diagnosis, improvement or treatment of physical and mental illness”.

Several studies have reported the capacity of TM in dry mouth symptoms, greatly referring to the use of herbal compounds (Table 2). Results are controversial and difficult to interpret, due to the differences in methods used in the studies [61].

#### 2.7.1. Traditional East Asian Medicine

The oldest use of herbal medicines belongs to traditional East Asian medicine. Based on its teaching, diseases are identified on the theories of Yin and Yang, related to the 5 elements and visceral manifestation theory. Herbal medicine is prescribed from pattern identification (or syndrome differentiation), based on the aforementioned theory, in addition to the patients’ conditions [69]. On these bases, herbal Chinese medicines were applied toward symptoms that could be related to xerostomia, and several trials have documented their behavior. Nevertheless, it is not possible to analyze and compare the clinical data, as the studies are often reported in Asian languages and only an English abstract is available on Scopus.

Among the few studies in English, several investigations pointed to reports of the efficacy of medicinal plants in treating oral mucositis, which often develops in a consequence of the oxidative stress and inflammation accompanying radiotherapy and chemotherapy treatments. Different plants have been indicated as suitable. One of them is Plantago major L. (*Greater plantain*), an herbaceous perennial plant, greatly used, due to its wound-healing properties, and anti-ulcerative, anti-inflammatory, anti-bacterial, anti-viral, and antioxidant activities [70,71]. Japanese traditional medicine *Hangeshashinto* (*Kampo*), which is a mixture of different radices, rhizomes, and fruits [72], was also reported as an effective agent in oral inflammation, due to its capacity in inhibiting the COX2 enzyme [73]. *Matricaria recutita* L. (*Chamomile*) was further investigated as a promising agent in the therapy of oral mucositis and recurrent aphthous stomatitis [74,75]. Additionally, the rhizome *Zingiber officinale* Roscoe (*Ginger*) was described in the relief of 5-fluorouracil-induced oral ulcerative mucositis and pain [76]. Also, the flavonoid quercitin, which enriches a large variety of herbs and fruits, has been indicated in the prevention of mucositis, because of its high anti-inflammatory and antioxidant potency [62]. Further, the capacity of *Malva sylvestris* L. (Mallow) and *Alcea digitata* (Boiss.) Alef was evaluated in a clinical trial within a 4-week period [63]. Using the VAS scale, the authors reported a significant efficacy of the medicinal herbs in comparison to artificial saliva. In one other comparative study, Hsu and colleagues [64] reported the relief of different Chinese herbs in antagonizing the symptoms suffered from patients undergoing radiotherapy for HNC. The herbs were administered during radiotherapy in the form of decoctions, topical applications, and/or powders, according to the symptoms emerging. The subjective perception of dry mouth and the objective findings recorded by clinicians were less severe in the case group than in the control cohort, even if the data were not significant.

The common use of herbal mixtures against oral diseases was reported by Park and colleagues [65,69]. The five most-used preparations were Shennongbaijie decoction, Xuanmaizengyehuadu decoction, Yunnanbaiyao capsules, Niancianchuanqipipa gel, Jiaweizengye decoction, and Sanganhuayin decoction. Furthermore, a great variety of medicinal herbs were mentioned as useful by Nabil and colleagues [77].

The clinical studies evaluating the efficacy of East Asian TM against symptoms emerging during radiotherapy for HNC are in greater numbers in comparison to those conducted using the Western TM. However, a very-low-to-moderate quality of evidence emerged from those studies [77]. In fact, they have high risk of bias in blinding of participants and personnel, incomplete outcome data, reported inaccurate results, or omitted participant characteristics. Information on randomization sequencing, allocation concealment, blinding, and dropouts were deficient. Although there was a potential improvement in salivary gland function, the methodological limitations reduced the strength of the evidence [61]. Consequently, more modeling studies are needed to better understand the effectiveness of these treatments.

#### 2.7.2. Traditional Western Countries’ Medicine

Like East Asian TM, any popular medicine in Western countries can refer to the treatment of xerostomia, but also to a series of symptoms, which, according to the current knowledge, could be included in dry mouth [78,79]. Symptoms have been mostly treated by applying a mixture of plants or herbs, or by using the common means of water extraction with multiple oral administration of infusions or decoctions [80]. However, given the scarcity of writings with which TM was handed down, the application of knowledge in the medical field is lacking. This issue has led to a large gap in information, considering the increasing number of patients who are integrating TM into conventional therapy [81]. In fact, several European research [82,83,84] evidenced that 15–73% of cancer patients, also suffering from low QoL, are adopting TM as an auto-medication to counteract the side effects of radio- and chemotherapies [85]. In view of this, the European Medicines Agency (EMA) and the Committee on Herbal Medicinal Products (HMPC) have established guidelines to recognize the pharmaceutical ability of herbs. A well-established medical use, dating back more than 30 years, and the absence of documented toxicity for more than 15 years, represent the keystones to consider popular remedies in a pharmacological field.

In this contest, several studies have been conducted in Europe, with the intent to validate herbal medicines, but a great number of them have been carried out using laboratory methods, instead of clinical evaluations. Very few trials have been conducted avoiding any oral systemic administration recommended by tradition, so limiting the efficacy of herbs as mouthwashes based on water extracts. This is the case for the instances of *Thymus vulgaris* L. (*thyme*) and honey [66], and a mixture of propolis, *Aloe vera* (L.) Burm. f. (*aloe-vera*), *Calendula officinalis* L. (*Pot marigold*), and *Matricaria recutita* L. [67], both applied as a mouthwash in the prevention of oral diseases in cancer patients. *Salvia officinalis* L. (*sage*) was studied as a spray against acute pharyngitis [86] and oral mucositis [68]; Echinacea/sage spray was also assayed against sour trough [87], as well as *Alchemilla vulgaris* [88]; and a mixture of *Matricaria recutita* L., *Calendula officinalis* L. and *Aloe vera* L., in addition to honey, was also studied [89].

Conversely, the capacity of different doses of essential oils or extracts of herbs have been widely studied in Europe by in vitro or in cell line models. Several research focused exclusively on the capacity of the materials to antagonize inflammation and oxidate stress [90,91]. Particularly, the power of *Malva sylvestris*, *Calendula officinalis* L., and *Matricaria recutita* L. was proven [92,93,94]. Also, the antimicrobial capacity ofmedicinal herbs has been extensively investigated in Europe. The sensibility of laboratory and clinical spp. of *Candida albicans* to *Thymus capitatus* (L.) Hoffmanns. & Link (*conehead thyme*), *Pistacia lentiscus* L. (*lentisk*), and propolis was examined, reporting great toxicity of the yeast [80,95,96]. As well, *Pistacia terebinthus* ssp. *terebin Pistacia terebinthus* L. subsp. *terebinthus* L (*terebinth*), *Cytinus hypocistis* (L.) L., and *Limonium morisianum* Arrigoni emerged as promising antimicrobials toward reference strains causing septicemia in compromised patients also suffering from mucositis [97]. However, even if in vitro and cell line systems are largely recommended to elucidate any herb, the same should not exert in vivo the behavior showed in bench [98,99]. Additional limits to prove the efficacy of TM in Western countries are represented by the need to characterize the pharmacodynamic and pharmacokinetic properties of any single, active compound composing the herb [100].

In a consequence of the above-mentioned considerations, and according to the international quality standards, the insufficient number of clinical evaluations represent the greatest limitation to prove the efficacy and safety of TM in Europe, and then, to validate any compound against xerostomia [85].

## 3. Discussion

In this overview, the most recent 10 years of literature regarding advancement in preventing and treating xerostomia using systemic chemical drugs and TM was reviewed. As a result of this research, it can be said that, regardless of the great number of agents evaluated in the trials, up to today, no compound has reached the goal to prevent or resolve xerostomia.

Preventing xerostomia in predisposed patients mainly concerns patients suffering from HNC. They end the radiotherapy fractions with xerostomia as a secondary effect of the oxidative process induced by radiation though the salivary glands, even if Intensity Modulated Radiotherapy (IMRT) is applied [101]. Amifostine acts as a free radical scavenger, DNA protective, and acceleration repair agent, also inducting cellular hypoxia. These mechanisms may antagonize salivary gland cell dysfunctions under radiation therapy. Based on the clinical data, several studies suggest the capacity of the drug in preventing the feeling of dryness after radiotherapy. Also, studies discuss whether amifostine has an influence on overall survival, progression-free survival, disease-free survival, or locoregional tumor control. Despite the positive considerations, the great number of side effects reduce the use of amifostine, currently.

Another class of discussed protective agents against xerostomia is the antioxidants group. These agents act directly against the oxidative stress as key factors in development of the disease in radiotherapy patients and subjects in need of chemotherapy. Further, antioxidants seem to be important tools in the cases of Sjögren Syndrome and systemic sclerosis, which are accompanied by a disruption of the oxidative eustress, strictly related to the progression of salivary gland failure. CoQ10 and Vitamin E, sometimes associated with Vitamin C, together with a great number of natural carotenoids, have been evaluated to counteract a decreased capacity of antioxidant systems, in view of increasing ROS. In general, the use of antioxidants has proved their efficacy by a greater increase in the salivary fluid in comparison to the baseline value. The data is thought to be a consequence of the whole recovery of salivary gland functions, due to the antioxidant activity. However, no detailed guidelines have been reported to adopt the compounds in the prevention of dry mouth.

Concerning the evidence of xerostomia, pilocarpine is one of the most assayed para-sympathomimetic drugs in clinical trials. With the intent to increase salivary output, pilocarpine has been largely applied in HNC and Sjögren’s Syndrome patients, who are the subjects who suffer most from a lack of saliva. Although a reliever of dry mouth in HNC patients, statistically significant evidence is very confusing when pilocarpine is compared to the placebo groups. Conversely, the important side effects of the drug really emerged in clinical trials. Further para-sympathomimetic drugs include cevimeline hydrochloride and bethanechol. Similarly, their administration resulted in low capacity in relieving dry mouth, and in a great number of side effects. Headache, urinary frequency, vasodilatation, dizziness, dyspepsia, nausea, asthenia, and diarrhea are recurrent clinical signs of the cholinergic activity of the drugs’ administration against xerostomia. All these express the fact that parasympathomimetic drugs stimulate indifferently the five types of mAChRs, and there is the need for a selective stimulant for salivary output.

In Sjögren’s patients, biological agents have been greatly experimented in the relief of dry mouth. In this field, rituximab, epratuzumab, belimumab, ianalumab, and baminercept are monoclonal antibodies used to antagonize the lymphocytic infiltration, which results in xerostomia and keratoconjunctivitis *sicca*. However, the use of these drugs has shown low efficacy in conjunction to possible great toxicity, ranging from allergic reactions to systemic infections. The same result has been obtained by administering interferon alpha, whose gastrointestinal effects really restrict its use, despite the inflammatory and immune response regulation.

Non-pharmacological therapy includes traditional or indigenous forms of healing, which are firmly rooted in the culture and history of populations. In Asian and Chinese TM, Shennongbaijie decoction, Xuanmaizengyehuadu decoction, Yunnanbaiyao capsules, Jiaweizengye decoction, and Sanganhuayin decoction represent commonly applied mixtures against oral dryness and mucositis. The preparations have different formulations, but all of them are taken as oral systemic compounds. A great variety of medicinal herbs are also indicated as able to manage oral ulcers and mucositis, deriving from dry mouth. Among them, *Hangeshashinto* (*Kampo*), *Plantago major*, *Matricaria recutita, Zingiber officinale*, *Malva silvestris*, and *Alcea digitate* are recurrent agents used in Asian TM. However, very-low-to-moderate quality of evidence has been reported in the current studies thus reducing the strength of evidence.

In Western countries, many herbs or mixtures have been reported as remedies towards oral infections, oral ulcers, cough, sore throat, and anxiety, all of which are included in dry mouth symptoms. As in Asian TM, in Europe, these remedies have been generally based on mixtures of plants or herbs orally administered as water extractions, in the form of infusions or decoctions. In this regard, the most utilized herbs have been *Malva sylvestris*, *Thymus vulgaris*, *Calendula officinalis L.*, *Matricaria recutita* L, *Salvia officinalis,* and propolis. However, a great number of studies in European countries have been carried out in vitro or in cell line models. Conversely, clinical evaluations against xerostomia are currently lacking. Furthermore, few clinical trials have been conducted modifying the popular background of use, due to restrictions imposed by the European Ethical Committees. An example of this is represented by the quite exclusive administration of water extracts as mouthwashes, instead of oral beverages. Additional limits are represented by the continued research to isolate the active compounds in herbal mixtures, characterizing their pharmacodynamic and pharmacokinetic properties. These intents are not in accordance with the multidimensional complexity of the natural compounds, where synergic effects between the different fractions are extremely important in the results, independently by the concentration of each biomolecule.

In conclusion, it can be said that xerostomia lacks drugs able to prevent the development of the disease in predisposed patients, currently. Furthermore, there is no systemic chemical treatment indicated for the relief of the disease in clinical practice. Regarding TM, the very low number of clinical studies does not allow for scientific validation of popular remedies. Consequently, the management of the disease and its devasting complications remain a very significant void in daily clinical practice.

## Figures and Tables

**Table 1:** Pharmacological systemic preparations used in the relief of xerostomia.

Reference	Experimental Preparation	Vector	Sample Characteristics	Time Treatment	Sialometry	Assessment	Time Points Assessment	Results
[19]	Pilocarpine vs. bromhexine	Tablets	25 HNC patients with radiotherapy-induced xerostomia	2 weeks	UWS	Xerostomia inventory questionnaire	Baseline vs. 2 weeks	Pilocarpine significantly improved saliva vs. bromhexine.
[20]	Pilocarpine vs. placebo	Bottled solution	11 HNC patients undergoing radiotherapy	5 weeks	UWS, WSS	WHO criteria	Baseline vs. 1, 2, 3, 4, 5-week controls	Pilocarpine increased saliva. Xerostomia and complication lowered.
[21]	Cevimeline vs. placebo	Capsules	54 HNC patients after radiotherapy	6 weeks	Not assessed	OHIP-49, QoL	Baseline vs. 6-week controls	No difference reported.
[22]	Bethanecol vs. placebo	Tablets	97 HNC patients with radiotherapy-induced xerostomia	From the beginning to 1 month after radiotherapy completion	UWS, WSS	Observer-based grade and scores according to subjective measures	Baseline vs. 3 months after the treatment	Bethanechol significantly improved salivary parameters.
[23]	Ubiquinol, Ubiquinone vs. placebo	Capsules	20 Sjogren vs. 22 healthy patients	4 weeks	WSS	Mental, physical, and oral conditions questionnaire	Baseline vs. 4-week control	Ubiquinone and ubiquinol increased significantly saliva. Questioner parameters significantly improved vs. control.
[24]	Ubiquinol vs. placebo	Gummy Candy	40 healthy patients suffering from xerostomia	8 weeks	WSS	Mental, physical, and oral conditions questionnaire	Baseline vs. 8-week control	WSS significantly improved vs. control.
[25]	Vitamin E + Vitamin C vs. placebo	Pills	23 HNC patients undergoing radio therapy	12 weeks	Not assessed	VAS scale	Baseline vs. 1- and 6-months after radiotherapy	Vit E + C lowered xerostomia

Acronyms: VAS = Visual Analogue Scale for xerostomia; HNC = Head and Neck Cancer; OHIP-14 = 14 items Oral Health Impact Profile; QoL = Quality of Life; WSS = Whole Stimulate Saliva; UWS = Unstimulated Whole Saliva.

**Table 2:** Herbal preparations used in the relief of xerostomia.

Reference	Experimental Preparation	Vector	Sample Characteristics	Time Treatment	Sialometry	Assessment	Time Points Assessment	Results
[62]	Quercetin hydrate vs. placebo	Capsules	20 patients under chemotherapy	From the beginning of radiotherapy up to the 4-week	Not assessed	WHO criteria	Baseline vs. daily control up to the completion of chemotherapy	No differences were reported.
[63]	Herbal compound (*Malva sylvestris* and *Alcea digitata*) vs. artificial saliva *(Hypozalix*)	Mouth rinse	62 irradiated patients	From the beginning of radiotherapy up to the 4-week	Not assessed	VAS	Baseline vs. 2- and 4-week controls	Xerostomia was significantly reduced vs. control.
[64]	Gan Lu Yin (unspecified composition) vs. control	Powder	91 HNC patients under radiotherapy/chemoradiotherapy	From the beginning of radiotherapy up to the 6-week	Not assessed	RTOG criteria	Baseline vs. 4, 7-week control, and at the completion of radiotherapy	Xerostomia was reduced, QoL improved significantly vs. control.
[65]	Jiaweizengye (Glycyrrhizae *Radix, Trichosanthis Radix, Scrophulariae* *Radix, Liriopes Radix, Adenophorae Radix, Dendrobii Herba, Mume Fructus, Puerariae Radix, Rehmanniae* *Radix*) vs. human epidermal growth factor	Decoction	60 HNC patients under radiotherapy	From the beginning to radiotherapy completion	WSS	RTOG criteria	Baseline vs. the completion of radiotherapy	WSS improved significantly. Xerostomia was lowered vs. control.
[66]	Thyme honey vs. saline solution	Mouth rinse	72 HNC patients under radiotherapy or/and chemotherapy	From the beginning of radiotherapy up to the 4-week after completion	Not assessed	NCI; QoL, VAS	Baseline vs. 1 and 6 months after the completion of radiotherapy	QoL improved significantly.
[67]	Faringel (*Propolis powder extract 6%, Aloe vera 30%, Calendula powder extract 2%, Chamomile aqueous solution 0,3%, Honey, Sodium alginate, Sodium Carbonate)* vs. placebo	Mouth rinse	107 HNC patients under radiotherapy	From the beginning of radiotherapy up to the 5-week	Not assessed	CTCAE	Baseline vs. weekly controls up to the completion of the radiotherapy	No differences were reported.
[68]	Traumeel S (*Arnica montana, Calendula officinalis, Achillea millefolium, Chamomilla recutita, Symphytum officinale, Atropa belladonna, Aconitum napellus, Bellis perennis, Hypericum perforatum, Echinacea angustifolia, Echinacea purpurea, Hamamelis virginica, Mercurius solubilis,* and *Hepar sulfuris*) vs. sage tea	Mouth rinse	20 HNC patients under radiotherapy or radio-chemotherapy	From the beginning to the radiotherapy completion	Not assessed	QLQ-C30; H&N35, CTCA, patients’ diaries, taste perceptions; oral inspection	Baseline vs. the completion of the treatment	No differences were reported.

Acronyms: VAS = Visual Analogue Scale for xerostomia; HNC = Head and Neck Cancer; QoL = Quality of Life; WSS = Whole Stimulate Saliva; H&N35 = Head and Neck questionnaire; CTCAE = Common Terminology Criteria for Adverse Events; NCI = National Cancer Institute scale; RTOG = Radiation Therapy Oncology Group criteria.
